# Walking as an Opportunity for Cardiovascular Disease Prevention

**DOI:** 10.5888/pcd16.180690

**Published:** 2019-05-30

**Authors:** John D. Omura, Emily N. Ussery, Fleetwood Loustalot, Janet E. Fulton, Susan A. Carlson

**Affiliations:** 1 Division of Nutrition, Physical Activity, and Obesity, National Center for Chronic Disease Prevention and Health Promotion, Centers for Disease Control and Prevention, Atlanta, Georgia; 2 Division for Heart Disease and Stroke Prevention, National Center for Chronic Disease Prevention and Health Promotion, Centers for Disease Control and Prevention, Atlanta, Georgia

## Abstract

**Introduction:**

Cardiovascular disease (CVD) is the leading cause of death in the United States, and increasing physical activity can help prevent and manage disease. Walking is an easy way for most adults to be more active and may help people at risk for CVD avoid inactivity, increase their physical activity levels, and improve their cardiovascular health. To guide efforts that promote walking for CVD prevention and management, we estimated the prevalence of walking among US adults by CVD risk status.

**Methods:**

Nationally representative data on walking from participants (N = 29,742) in the 2015 National Health Interview Survey Cancer Control Supplement were analyzed. We estimated prevalence of walking (ie, any, transportation, and leisure) overall and by CVD status. We defined CVD status as either not having CVD and not at risk for CVD; being at risk for CVD (overweight or having obesity plus 1 or more additional risk factors); or having CVD. We defined additional risk factors as diabetes, high cholesterol, or hypertension. Odds ratios were estimated by using logistic regression models adjusted for respondent characteristics.

**Results:**

Prevalence of any walking decreased with increasing CVD risk (no CVD/not at risk, 66.6%; at risk: overweight or has obesity with 1 risk factor, 63.0%; with 2 risk factors, 59.5%; with 3 risk factors, 53.6%; has CVD, 50.2%). After adjusting for respondent characteristics, the odds of any walking and leisure walking decreased with increasing CVD risk. However, CVD risk was not associated with walking for transportation.

**Conclusions:**

Promoting walking may be a way to help adults avoid inactivity and encourage an active lifestyle for CVD prevention and management.

**Summary**

**What is already known on this topic?**

Increasing physical activity among adults at risk for or with cardiovascular disease (CVD) can help prevent and manage the disease, and walking is an easy way for most adults to avoid inactivity and increase physical activity levels. However, the prevalence of walking among people at various degrees of CVD risk is unknown.

**What is added by this report?**

The prevalence of any walking decreases with increasing CVD risk, even after adjusting for demographic characteristics. Similar patterns are observed for leisure walking but not for transportation walking.

**What are the implications for public health practice?**

Promoting walking, especially among adults at high risk, may present an important opportunity for encouraging active lifestyles for CVD prevention and management.

## Introduction

Cardiovascular disease (CVD) is the leading cause of death in the United States, and physical inactivity is an important modifiable risk factor (1). Increasing physical activity among adults at risk for or with CVD can help prevent and manage disease (1). The *Physical Activity Guidelines for Americans*, second edition (Guidelines), suggests that regular physical activity can help improve quality of life for people with chronic health conditions and reduce their risk of developing new conditions (2). The Guidelines recommend that adults with chronic conditions be physically active on a regular basis: adults with chronic conditions who are able should do at least 150 to 300 minutes of moderate-intensity aerobic physical activity a week or 75 to 150 minutes of vigorous-intensity activity or an equivalent combination of both (2). The review of scientific evidence supporting the Guidelines affirmed a well-established relationship between regular physical activity and cardiovascular health (3,4). Everyone can gain cardiovascular health benefits from physical activity (4). Some physical activity is better than none, and more physical activity is even better (2).

Recognizing the benefits of healthy behaviors, including physical activity for CVD prevention, the US Preventive Services Task Force (USPSTF) recommends that health care providers offer or refer adults who are overweight or have obesity and have additional CVD risk factors to intensive behavioral counseling interventions to promote a healthful diet and physical activity for CVD prevention (5). More than 1 in 3 US adults is considered part of this at-risk population, and almost 1 in 5 is at risk and does not meet the aerobic component of the Guidelines (2,6). Walking has been associated with meeting the aerobic component of the Guidelines (2,7). Walking is an easy way for most adults to initiate or increase physical activity in their daily routines (8). Consequently, walking may present an opportunity for promoting physical activity among those at high risk for CVD, offering a simple way to avoid inactivity and increase physical activity.

Physical activity, including walking and other forms of active transportation, promotes cardiovascular health (3). Previous studies showed that adults with CVD are less likely to be physically active than healthy adults (9–11), although to our knowledge no study has examined walking as a form of physical activity. In addition, previous studies have largely demonstrated the positive effect of walking and active transportation on improving individual cardiovascular risk factors such as hypertension, body mass index (BMI, weight in kilograms divided by height in meters squared), and diabetes as well as cardiovascular disease end points such as incident coronary heart disease, stroke, and death (12–14). However, to our knowledge, no study has examined the prevalence of walking among people at increasing levels of cardiovascular risk and disease. To provide health care providers with information about the prevalence of walking among US adults by CVD risk status, the objectives of this study were 1) to estimate the national prevalence of walking, including different types of walking, among US adults at discrete levels of cardiovascular risk and disease; and 2) to examine the association between the degree of cardiovascular risk and disease with any walking and with walking for leisure and transportation. We examined walking for leisure and transportation separately because previous research has demonstrated that different types of walking have unique facilitating factors and associated barriers (8,15–18). Thus, understanding which types of walking are more or less prevalent in these populations can help to inform future interventions.

## Methods

### Study sample

The National Health Interview Survey (NHIS) is a continuous, cross-sectional survey of US households representative of the civilian, noninstitutionalized population and is administered by in-person interviews (19). NHIS consists of a core questionnaire that collects basic health and demographic information for all family members in a sampled household and periodic questionnaire supplements that address special topics. Questions on walking for leisure and transportation were asked of 1 randomly selected adult aged 18 or older per sampled household in the 2015 NHIS Cancer Control Supplement. The sample adult response rate was 55.2%.

From the initial total sample of 33,672, respondents were excluded if they were missing data on walking (1,916), health-related characteristics (1,000), or demographic characteristics (172). In addition, respondents who reported being unable to walk were excluded (842). The final analytic sample was 29,742 adults.

### Measures

Transportation walking was defined as a yes response to the question, “During the past 7 days, did you walk to get someplace that took you at least 10 minutes?” Leisure walking was defined as a yes response to the question, “During the past 7 days, did you walk for at least 10 minutes [for fun, relaxation, exercise, or to walk the dog]?” Any walking was defined as participating in either transportation or leisure walking.

We assessed sex, age, race/ethnicity, education level, region of residence, current smoking status, BMI category, and hypertension, hyperlipidemia, diabetes, myocardial infarction, and stroke status. BMI was calculated for each respondent on the basis of self-reported weight and height. Respondents were categorized as underweight or normal weight (BMI <25), overweight (BMI 25–<30), or having obesity (BMI ≥30) (20). Respondents were defined as having hypertension, hyperlipidemia, diabetes, myocardial infarction, or stroke if they responded yes to questions asking if they had ever been told by a doctor or other health professional that they had hypertension (also called high blood pressure), high cholesterol, diabetes or sugar diabetes (other than during pregnancy), heart attack (also called myocardial infarction), or stroke.

Respondent characteristics were used to categorize respondents into various categories based on CVD status. Respondents were defined as having CVD if they had a reported history of stroke or myocardial infarction. They were defined as being at risk of CVD if they were overweight or had obesity and had 1, 2, or 3 additional risk factors defined by the USPSTF (hypertension, hyperlipidemia, or diabetes) (5). Respondents who fell into neither of these categories were defined as having no CVD and not at risk for CVD.

### Statistical analysis

Prevalence and 95% confidence intervals (CIs) of walking (any, transportation, and leisure) were examined overall, by CVD status, and by respondent characteristics. Adjusted Wald tests, pairwise *t* tests, and orthogonal polynomial contrasts were used to identify significant differences and trends where appropriate. Logistic regression analyses adjusting for respondent characteristics (ie, sex, age group, race/ethnicity, education, region of residence, and current smoking status) were conducted to examine the odds of any, leisure, and transportation walking by CVD status. In addition, analyses were conducted to assess trends limited only to those without CVD. Sampling weights provided by the National Center for Health Statistics were applied to produce nationally representative estimates, and results were deemed significant at *P* < .05. We performed analyses in 2018 by using SUDAAN Version 11.0 (Research Triangle Institute) to account for the complex sample design.

## Results

Our sample was 51% women, and 53% were aged 45 or older. Most were non-Hispanic white (65%) and had at least some college education (63%), and the largest proportion were in the South Census region (37%) (Table 1). Most (67%) adults had no CVD and were not at risk. The prevalence of adults at risk who were overweight or had obesity with 1 risk factor was 15.7%; with 2 risk factors, 9.1%; and with 3 risk factors, 3.4%. The prevalence of adults with CVD was 4.7%.

**Table 1:** Sample Characteristics and Prevalence of Walking Among US Adults, National Health Interview Survey, 2015^a^

Characteristic^b^	Sample Size^c^ (%)^d^	Type of Walking, % (95% Confidence Interval)		
		Any	Leisure	Transportation
**Total**	29,742 (100)	64.2 (63.3–65.1)	53.0 (52.1–54.0)	32.6 (31.7–33.4)
**Sex**				
Male	13,618 (49.4)	63.6 (62.4–64.9)	51.2 (50.0–52.5)	35.3 (34.1–36.6)
Female	16,124 (50.7)	64.7 (63.6–65.8)	54.8 (53.6–56.0)	29.9 (28.8–30.9)
**Age, y**				
18–24	2,673 (12.8)	65.7 (62.9–68.4)	49.5 (46.7–52.2)	41.0 (38.0–44.0)
25–34	5,223 (17.9)	67.7 (65.9–69.4)	56.4 (54.5–58.2)	35.1 (33.3–37.0)
35–44	4,743 (16.7)	66.0 (63.9–68.0)	55.0 (52.9–57.1)	33.5 (31.6–35.5)
45–64	9,954 (34.1)	64.4 (63.0–65.9)	53.8 (52.3–55.4)	31.5 (30.1–32.8)
≥65	7,149 (18.5)	57.5 (55.9–59.1)^e^	49.0 (47.4–50.6)^f^	25.4 (24.0–26.8)^g^
**Race/ethnicity**				
White, non-Hispanic	18,565 (65.2)	64.9 (63.8–66.0)	55.0 (53.8–56.1)	31.0 (29.9–32.1)
Black, non-Hispanic	3,815 (11.4)	57.7 (55.3–60.1)	43.7 (41.3–46.2)	35.0 (32.7–37.3)
Hispanic	4,951 (15.7)	62.7 (60.8–64.5)	49.6 (47.7–51.4)	34.5 (32.6–36.4)
Other	2,411 (7.7)	70.5 (68.0–72.8)	57.5 (55.0–60.1)	38.1 (35.4–40.9)
**Education**				
Less than high school diploma	3,968 (12.2)	53.9 (51.8–56.0)	41.6 (39.4–43.8)	32.4 (30.4–34.4)
High school diploma	7,365 (24.5)	56.4 (54.7–58.0)	46.0 (44.2–47.7)	27.0 (25.5–28.4)
Some college	9,292 (31.3)	63.3 (61.9–64.8)	51.6 (50.1–53.1)	31.0 (29.6–32.5)
College graduate	9,117 (32.0)	74.9 (73.6–76.1)^e^	64.1 (62.7–65.5)^e^	38.4 (36.9–40.0)^e^
**Region**				
Northeast	4,872 (17.2)	67.2 (65.2–69.1)	51.6 (49.3–54.0)	41.0 (38.9–43.2)
Midwest	6,275 (22.3)	62.2 (60.4–63.9)	52.3 (50.4–54.1)	29.5 (27.8–31.2)
South	10,172 (37.0)	59.7 (58.1–61.3)	50.1 (48.5–51.7)	27.4 (25.9–29.0)
West	8,423 (23.5)	70.9 (68.9–72.7)	59.3 (57.4–61.3)	37.4 (35.6–39.1)
**Current smoker**				
Yes	4,784 (14.9)	55.9 (54.0–57.8)	44.1 (42.2–46.0)	30.6 (28.8–32.4)
No	24,958 (85.1)	65.6 (64.7–66.5)	54.6 (53.6–55.6)	32.9 (32.0–33.8)
**Body mass index category^h^**				
Underweight or normal weight	10,521 (36.4)	68.3 (67.0–69.6)	56.5 (55.1–57.8)	36.4 (35.0–37.9)
Overweight	10,128 (33.8)	64.2 (62.8–65.5)	53.7 (52.3–55.1)	31.0 (29.8–32.2)
Has obesity	9,093 (29.8)	59.2 (57.7–60.6)	48.0 (46.6–49.5)	29.6 (28.2–30.9)
**Diabetes**				
Yes	3,563 (10.7)	54.2 (51.9–56.4)	44.0 (41.8–46.3)	25.6 (23.7–27.6)
No	26,179 (89.3)	65.4 (64.4–66.3)	54.1 (53.1–55.1)	33.4 (32.4–34.4)
**Hyperlipidemia**				
Yes	8,575 (27.2)	61.6 (60.3–63.0)	51.3 (49.8–52.8)	29.6 (28.3–30.9)
No	21,167 (72.8)	65.1 (64.1–66.1)	53.7 (52.6–54.8)	33.7 (32.6–34.7)
**Hypertension**				
Yes	8,857 (26.2)	57.9 (56.5–59.3)	48.0 (46.5–49.5)	27.7 (26.4–29.1)
No	20,885 (73.8)	66.4 (65.4–67.4)	54.8 (53.7–55.9)	34.3 (33.2–35.4)
**Myocardial infarction**				
Yes	1,027 (2.9)	52.0 (47.8–56.2)	43.4 (39.4–47.5)	22.2 (18.9–25.9)
No	28,715 (97.1)	64.5 (63.6–65.4)	53.3 (52.4–54.3)	32.9 (32.0–33.8)
**Stroke**				
Yes	897 (2.4)	47.0 (42.5–51.6)	37.8 (33.5–42.2)	23.2 (19.7–27.0)
No	28,845 (97.6)	64.6 (63.7–65.5)	53.4 (52.4–54.4)	32.8 (31.9–33.7)

a Excludes respondents unable to walk (n = 842).

b All characteristics were significantly associated with the prevalence of any, leisure, and transportation walking (*P* < .05 based on adjusted Wald tests), except for the association between any walking and sex (*P* = .18).

c Sample sizes are unweighted.

d Percentages are weighted and may not add to 100% because of rounding.

e Significant linear and quadratic trends (*P* < .05). Demonstrates nonlinear variation in addition to an overall increase or decrease.

f Significant quadratic trend (*P* < .05).

g Significant linear trend (*P* < .05).

h Body mass index (weight in kilograms divided by height in meters squared) estimates were calculated from self-reported weight and height. Underweight and normal weight = BMI <25, overweight = BMI 25.0–29.9, and has obesity = BMI ≥30.

Overall, the prevalence of walking was 64% for any walking, 53% for leisure walking, and 33% for transportation walking (Table 1). Significant differences in prevalence of any, leisure, and transportation walking were observed for all characteristics, except for any walking by sex. In addition, prevalence of any, leisure, and transportation walking decreased linearly with increasing BMI and was lower among adults with diabetes, hyperlipidemia, hypertension, myocardial infarction, and stroke than among those without. In unadjusted analyses, the prevalence of any, leisure, and transportation walking decreased linearly with increasing CVD risk (Figure). For example, the prevalence of any walking among adults who had no CVD and were not at risk was 66.6%; for those who were overweight or had obesity with 1 risk factor, 63.0%; for those with 2 risk factors, 59.5%; for those with 3 risk factors, 53.6%; and for those with CVD, 50.2%.

**Figure:**
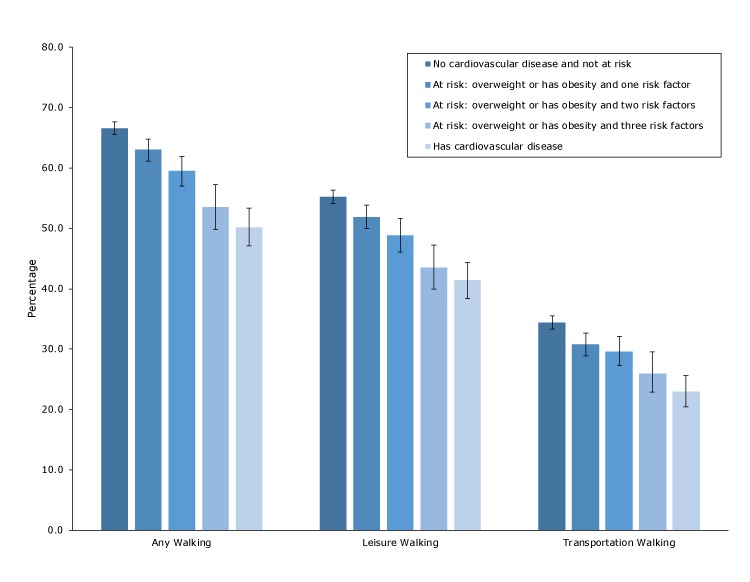
Prevalence of walking among US adults by cardiovascular disease status, National Health Interview Survey, 2015 (N = 29,742). Excludes respondents unable to walk (n = 842). Error bars represent the upper and lower bounds of the 95% confidence interval. Risk factors were hypertension, hyperlipidemia, or diabetes. Significant linear trends by cardiovascular disease status (*P* < .05) were observed for any walking, leisure walking, and transportation walking.

**Table:** 

Cardiovascular Disease Status	Type of Walking, % (95% Confidence Interval)		
	Any	Leisure	Transportation
No cardiovascular disease and not at risk	66.6 (65.6–67.6)	55.2 (54.1–56.3)	34.4 (33.3–35.5)
At risk: overweight or has obesity and 1 risk factor	63.0 (61.1–64.8)	51.9 (50.0–53.9)	30.8 (28.9–32.7)
At risk: overweight or has obesity and 2 risk factors	59.5 (57.0–61.9)	48.8 (46.1–51.6)	29.6 (27.3–32.1)
At risk: overweight or has obesity and 3 risk factors	53.6 (49.8–57.3)	43.5 (39.9–47.2)	26.0 (22.9–29.5)
Has cardiovascular disease	50.2 (47.1–53.3)	41.4 (38.4–44.4)	22.9 (20.4–25.6)

After adjusting for respondent characteristics, the association between any walking and leisure walking and CVD risk remained, and the adjusted odds of any walking and leisure walking decreased linearly with increasing CVD risk (Table 2). For example, when compared with adults with no CVD and not at risk, the adjusted odds ratio (AOR) of leisure walking among those who were overweight or had obesity and 1 risk factor was 0.87 (95% confidence interval [CI], 0.80–0.95); with 2 risk factors, AOR 0.81 (95% CI, 0.72–0.92); with 3 risk factors, AOR 0.72 (95% CI, 0.61–0.84); and with CVD, AOR 0.66 (95% CI, 0.58–0.76). These linear trends by CVD status were observed even after removing respondents with CVD. The adjusted odds of transportation walking also decreased linearly with increasing CVD risk; however, this trend was no longer significant after removing respondents with CVD. The adjusted odds of transportation walking was only lower among those with CVD compared with those with no CVD and not at risk (AOR, 0.74; 95% CI, 0.63–0.88).

**Table 2:** Adjusted Odds Ratios^a^ of Walking Among US Adults (N = 29,742), National Health Interview Survey, 2015^b^

Cardiovascular Disease Status^c^	Type of Walking, Adjusted Odds Ratio (95% Confidence Interval)		
	Any	Leisure	Transportation
No cardiovascular disease and not at risk	1 [Reference]		
At risk: overweight or has obesity and 1 risk factor	0.89 (0.81–0.97)	0.87 (0.80–0.95)	0.94 (0.84–1.04)
At risk: overweight or has obesity and 2 risk factors	0.84 (0.74–0.94)	0.81 (0.72–0.92)	0.97 (0.85–1.11)
At risk: overweight or has obesity and 3 risk factors	0.72 (0.61–0.85)	0.72 (0.61–0.84)	0.86 (0.72–1.03)
Has cardiovascular disease	0.65 (0.56–0.74)^d^	0.66 (0.58–0.76)^d^	0.74 (0.63–0.88)^d^

a Logistic regression model adjusted for sex, age group, race/ethnicity, education level, region of residence, and current smoking status.

b Excludes respondents unable to walk (n = 842).

c Cardiovascular disease status category definitions: 1) has cardiovascular disease (history of stroke or coronary heart disease); 2) at risk (overweight or had obesity and one or more additional risk factors [hypertension, hyperlipidemia, or diabetes]); and 3) no cardiovascular disease and not at risk (all others who did not fall into the other 2 categories).

d Significant linear trend (*P* < .05). Analyses were also conducted to assess trends limited only to those without cardiovascular disease. In these analyses, significant linear trends were still observed for any walking and leisure walking, but not for transportation walking.

## Discussion

Overall, only two-thirds of adults reported any walking for at least 10 minutes during the past 7 days. Our study found that the prevalence of any walking was lower among adults with higher degrees of CVD risk, and this association remained after adjusting for demographic characteristics. Although similar patterns were observed for leisure walking and CVD risk, they were not observed for transportation walking. Promoting walking, especially among adults at high risk, may present an important opportunity for encouraging an active lifestyle for CVD prevention and management.

Our study is unique in that we examined participation in walking among adults with discrete levels of CVD risk. A study using 2014 NHIS data examined the prevalence of adults who reported sufficient volume (≥150 minutes/week) of aerobic leisure-time physical activity by chronic disease status (9). This prevalence was found to be lower among participants with obesity, hyperlipidemia, hypertension, diabetes, myocardial infarction, and stroke than among apparently healthy adults (9). In addition, a study using data from the 2007–2008 and 2009–2010 cycles of the National Health and Nutrition Examination Survey found that engaging in active transportation was associated with more favorable cardiovascular risk factor profiles, including lower BMI, smaller waist circumference, and lower odds of hypertension and diabetes (21). Although these studies provide a general overview of physical activity among adults with or at risk for CVD, to our knowledge no study has examined walking as a source of physical activity among people with varying degrees of CVD risk. Our findings identify an opportunity for promoting walking as a form of physical activity among these high-risk groups. Future studies may also assess the effect of walking frequency and intensity and the interaction of other forms of aerobic and muscle-strengthening physical activity on the relationships observed in our study.

We observed decreasing odds of any walking and leisure walking with increasing CVD risk, which remained after adjusting for respondent characteristics. This finding is likely explained in part by the known relationship between low levels of physical activity and increased CVD and CVD risk (1). This inverse relationship identifies an opportunity to promote leisure walking among those at high risk for CVD to help them avoid inactivity and increase their participation in overall physical activity to prevent the development and progression of CVD. However, CVD risk was not associated with transportation walking, which was also the least common type of walking reported. These differing relationships by walking type may be due in part to distinct reasons for walking, such as participating in leisure walking to improve health or for personal interaction and social involvement (8). Future research investigating the underlying factors influencing these associations and how volume of walking differs by CVD risk (22) may help explain the various relationships we observed and inform effective strategies to promote walking for multiple purposes, especially among people at various levels of CVD risk.

Clinical interventions that promote physical activity can help prevent CVD. For example, the USPSTF recommends that health care providers offer or refer adults who are overweight or have obesity and have additional CVD risk factors to intensive behavioral counseling interventions to promote a healthful diet and physical activity for CVD prevention (5). More than 1 in 3 US adults is eligible for this intensive behavioral counseling, and almost 1 in 5 US adults is both eligible and does not meet the guideline for aerobic physical activity from the Guidelines (2,6). Despite this potential for a population-level health effect, compliance with this USPSTF recommendation remains low. In 2015, fewer than 1 in 10 primary care providers both discussed physical activity with most of their at-risk patients and referred them to intensive behavioral counseling (2,23). Primary care providers encounter several barriers to physical activity counseling (24–27), including a belief that patients will not participate in physical activity (23). However, walking may offer these providers a more appealing option for promoting physical activity among this high-risk group, because walking is an easy way for most adults to incorporate more physical activity into their daily routines (8). Additional research assessing providers’ attitudes toward walking and the uptake of walking among patients at risk for CVD would aid in understanding the potential effect of walking on improving physical activity among this high risk population.

Many strategies can be implemented in communities to promote walking that can benefit all adults, including those at risk for CVD. For example, communities can improve walkability by designing communities that make it safe and easy for all people to walk (8,15). In 2016, the Community Preventive Services Task Force recommended built environment approaches combining transportation system interventions with land use and environmental design to increase physical activity (28). In addition, various sectors can promote programs and policies to support walking where people live, learn, work, and play and provide information to encourage walking. Promoting walking at the community level may benefit everyone and can contribute to CVD prevention through increased physical activity.

This study has several limitations. Data were self-reported and may be subject to recall and social desirability biases (29). Data were also cross-sectional, making it difficult to rule out reverse causality. In addition, about 6% of survey respondents did not report on their walking behaviors. NHIS does not assess intensity of walking, and we were unable to assess whether the walking reported was brisk enough to offer substantial health benefits overall. Finally, the assessment of CVD risk relied on respondents reporting if they had ever been diagnosed with 1 of the disorders, and our assessment of walking was fairly broad, subjecting both to possible misclassification bias. Strengths of our study were the use of a large, national data set, which allowed us to generate representative estimates of walking behaviors among US adults with discrete levels of CVD risk. Also, the measures of walking examined in this study enabled assessment of both transportation and leisure walking.

Adults at different degrees of CVD risk participated at various levels in any walking and walking for leisure. In particular, the prevalence of any walking and leisure walking decreased as the degree of CVD risk increased. Our findings illustrate the potential of walking as a form of physical activity for CVD prevention. Findings can be used to inform clinical and community interventions that promote physical activity through walking for CVD prevention among those at various levels of CVD risk.
